# Genome size variation and evolution during invasive range expansion in an introduced plant

**DOI:** 10.1111/eva.13624

**Published:** 2023-12-11

**Authors:** F. Alice Cang, Shana R. Welles, Jenny Wong, Maia Ziaee, Katrina M. Dlugosch

**Affiliations:** ^1^ Department of Ecology and Evolutionary Biology University of Arizona Tucson Arizona USA; ^2^ Utah Valley University Orem Utah USA; ^3^ Mills College Oakland California USA

**Keywords:** flow cytometry, intraspecific genome size variation, invasive species, yellow starthistle

## Abstract

Plants demonstrate exceptional variation in genome size across species, and their genome sizes can also vary dramatically across individuals and populations within species. This aspect of genetic variation can have consequences for traits and fitness, but few studies attributed genome size differentiation to ecological and evolutionary processes. Biological invasions present particularly useful natural laboratories to infer selective agents that might drive genome size shifts across environments and population histories. Here, we test hypotheses for the evolutionary causes of genome size variation across 14 invading populations of yellow starthistle, *Centaurea solstitialis*, in California, United States. We use a survey of genome sizes and trait variation to ask: (1) Is variation in genome size associated with developmental trait variation? (2) Are genome sizes smaller toward the leading edge of the expansion, consistent with selection for “colonizer” traits? Or alternatively, does genome size increase toward the leading edge of the expansion, consistent with predicted consequences of founder effects and drift? (3) Finally, are genome sizes smaller at higher elevations, consistent with selection for shorter development times? We found that 2C DNA content varied 1.21‐fold among all samples, and was associated with flowering time variation, such that plants with larger genomes reproduced later, with lower lifetime capitula production. Genome sizes increased toward the leading edge of the invasion, but tended to decrease at higher elevations, consistent with genetic drift during range expansion but potentially strong selection for smaller genomes and faster development time at higher elevations. These results demonstrate how genome size variation can contribute to traits directly tied to reproductive success, and how selection and drift can shape that variation. We highlight the influence of genome size on dynamics underlying a rapid range expansion in a highly problematic invasive plant.

## 1 INTRODUCTION

Plants demonstrate some of the greatest variation in genome size among eukaryotes (Bennetzen, 2005) and the potential ecological and evolutionary consequences of this widespread variation remain an open question. At the most basic level, we expect that large genomes require greater cell volumes and longer replication times, and comparative analyses across plants bear out the generality of this relationship, along with associated reductions in stomatal density and increased seed mass (Beaulieu et al., 2008; Beaulieu, Leitch, & Knight, 2007). These differences suggest potential costs associated with maintaining large genomes, as greater cellular volumes and longer cell cycles might constrain functional traits related to growth, reproduction, and dispersal, including longer minimum generation times (Grotkopp et al., 2004), slower rates of photosynthesis (Knight, 2005), and smaller plant size (Carta & Peruzzi, 2016), among others (e.g., Francis et al., 2008; Kenton et al., 1986; Veselý et al., 2020). While there has been some success in identifying these effects at the cellular level and subsequent trait changes (Hodgson et al., 2010; Knight & Beaulieu, 2008), population consequences and fitness effects in particular environments remain less understood (Bilinski et al., 2018; Gaut & Ross‐Ibarra, 2008; Mei et al., 2017; Whitney et al., 2010).

Contrary to traditional understanding of genome size as a stable species‐level trait (e.g. Ohri, 1998), genome structure can vary dramatically across individuals and populations and over time, even on very short timescales. The predominant molecular mechanisms of genome expansion are whole genome or whole chromosome duplications, and transposable element (TE) proliferation within a ploidy level (Feschotte & Pritham, 2007; Lisch, 2013; Tenaillon et al., 2010). Conversely, unequal homologous recombination or illegitimate recombination drive losses of DNA content (Bennetzen, 2005). Some of the clearest evidence for the mutability of genome content and the processes that influence genome size has been observed in crop systems and wild crop relatives. Some of the earliest evidence emerged from studies of wild barley, in which variation in insertion patterns of a single family, BARE‐1, has contributed up to ~5% of genome size variation (Kalendar et al., 2000). In maize, the most common TE families that make up nearly ~70% of the genome have average insertion dates of less than 1 Mya, with some inserting as recently as 560 kya (Baucom et al., 2009). These recent bursts of TE proliferation are responsible for wide‐ranging genome size variation in maize, and repeat content explains a > 20% difference in genome size between the *Palomero* landrace and the B73 inbred line (Vielle‐Calzada et al., 2009). Likewise, differences in TE purging are strongly correlated with genome size variation in maize, where significant loss of TE content was observed after only a few generations in experimental maize lines (Roessler et al., 2019). Similar findings in flax have identified a comparable scale of variation, as well as inducible changes to genome size within one generation (Cullis, 2005), suggesting relatively few meiotic events are required to generate dramatic variation among closely related individuals. In cultivated rice, recent transposition of LTR‐RT transposable element families is responsible for genome size doubling, on the same scale of variation as whole genome duplications (Piegu et al., 2006). Work in select model systems has shown similar findings, including in *Arabidopsis* (Davison et al., 2007; Schmuths, 2004) and *Drosophila* species (Ellis et al., 2014; Petrov, 2003).

Few studies have been able to clearly attribute genome size variation to ecological and evolutionary processes. The *Arabidopsis* (Lockton et al., 2008; Lockton & Gaut, 2010) and *Drosophila* genetic model systems (González, Lenkov, et al., 2008; González, Macpherson, et al., 2008) have leveraged abundant sequence data and have identified signatures of both selection and drift in families of TEs known to contribute to variation in genome size. Again, crop systems also provide evolutionary insights, as in the genus *Oryza*, where TE variation was identified as the driver of divergence between different species, indicating that mechanisms generating genome size variation can also have important macroevolutionary implications (Zhang & Gao, 2017). In wild barley populations, BARE‐1 retroelement content varied across elevational and aridity gradients, suggesting an association with environmental conditions (Kalendar et al., 2000). Multiple systems have identified potential selective effects of elevation on genome size, potentially due to differences in growing season and the fitness effects of development time, as in maize (Bilinski et al., 2018), *Corchorus olitorius* (Benor et al., 2011), *Lagenaria siceraria* (Achigan‐Dako et al., 2008), as well as a small number of non‐crop systems such as *Dactylis glomerata* (Creber et al., 1994), and *Arachis duranensis* (Temsch & Greilhuber, 2001), though other results are conflicting (Lysák, 2000; Oney‐Birol & Tabur, 2018; Tuna et al., 2019). Taken together, these studies suggest that intraspecific genome size variation arises because the molecular mechanisms that influence genome size and subsequent structural variation are subject to the same evolutionary processes as other mutations. Subsequent accumulation of population‐level differences can be ecologically relevant and function as important sources of variation upon which selection can act. Yet, there is still a dearth of knowledge about the fitness effects of genome size variation in natural populations, and in non‐model systems.

Biological invasions present particularly useful natural laboratories to examine ecological consequences of intraspecific genome size variation and to infer selective agents that might drive genome size shifts. If genome size imposes developmental constraints, a potential invader may benefit from reduced genome sizes that promote fast generation time and high reproductive rates, which are traits associated with a colonizer life history (Baker, 1974). Thus, genome size‐associated traits that equip individuals to disperse, survive, and reproduce in marginal habitats may select for smaller genomes. In large multispecies comparisons, reported incidence of naturalization was more likely among species with smaller genomes, suggesting potential advantages during early stages of invasion (Pyšek et al., 2023). This may account for the overrepresentation of species with small genomes among weedy taxa in broad‐scale surveys of plant invaders globally (Kubešová et al., 2010; Pandit et al., 2014) and across the United States (Kuester et al., 2014). A comparative phylogenetic study of pines identified relationships between small genomes and “weedy” traits, such as small seed size, short minimum generation time, and fast relative growth rate, while also linking genome size to invasiveness as a character (Grotkopp et al., 2004). Importantly, invaded systems allow inter‐ and intra‐population comparisons of introductions with their source populations, to infer contemporary and rapid evolution of genome size. Direct comparisons between introduced populations and known sources are relatively few in number (Crosby et al., 2014; Pyšek et al., 2018) and methodological issues with genome size estimation can produce contested results (Lavergne et al., 2009; Martinez et al., 2018). Identifying evidence for selection on genome size during colonization requires systems in which the history of expansion is well‐documented, ecologically relevant traits are known, and robust genome size estimation methods are used.

In contrast to natural selection, neutral population genetic processes might also influence genome size variation. If processes such as TE proliferation generate genetic variants that are neutral or only weakly deleterious, genetic drift may allow sequences that increase average genome size to persist and spread. This is especially important in invading populations, where range expansion can diminish the effective population size of successive founding events at the leading edge (Braasch et al., 2019; Slatkin & Excoffier, 2012), and mutations arising in advancing populations can “surf” to higher frequency and broader geographic scales (Klopfstein et al., 2006; Gralka et al., 2016), even if these variants are disadvantageous (Peischl et al., 2013). Thus, if drift and founder effects are stronger than selection, we expect genome sizes to increase as an invasion expands across space.

Here, we investigate evolutionary causes of genome size variation in the invasive species yellow starthistle, *Centaurea solstitialis* L., in the Asteraceae (hereafter, YST). YST is an annual, obligately outcrossing, diploid thistle (2*n* = 16, Heiser & Whitaker, 1948; Öztürk et al., 2009; Widmer et al., 2007), and previous work has suggested that genome size is variable within its invaded ranges (Irimia et al., 2017). YST is invasive in the United States, where it is particularly problematic in California and invading populations are now distributed across more than 12 million acres of land (Maddox & Mayfield, 1985; Pitcairn et al., 2006). YST likely originated in the eastern Mediterranean before an ancient expansion into Eurasia and western Europe (Barker et al., 2017). Historical records indicate that it was introduced to South America from Spain and to the United States from Chile via imports of alfalfa, with the earliest recorded occurrence in the San Francisco Bay area in 1869 (Pitcairn et al., 2006). This recent colonization history has been confirmed by genetic studies (Barker et al., 2017; Dlugosch et al., 2013; Eriksen et al., 2014). We know from this previous work that Californian populations are derived from source populations in western Europe (Barker et al., 2017; Dlugosch et al., 2013) and have evolved shifts in growth rate and flowering time (Dlugosch et al., 2015), which are traits associated with genome size variation in other systems (e.g., Bilinski et al., 2018; Carta & Peruzzi, 2016; Grotkopp et al., 2004). We also know that the invasion has crossed large elevational and environmental gradients (Braasch et al., 2019; Gerlach, 1997; Pitcairn et al., 2006), which provide an opportunity to examine the influence of environmental clines on genome size variation, decoupled from invasion history. In particular, evidence from other plant systems (e.g., Achigan‐Dako et al., 2008; Benor et al., 2011; Bilinski et al., 2018) suggests higher elevations in particular may impose selection for smaller genome sizes.

We leverage understanding of the expansion history of YST in California to test predictions about how genome size variation might have evolved under different evolutionary processes. If large genome size constrains growth and reproduction as observed in other systems, we expect selection to favor smaller genome sizes during the process of range expansion, and at high elevations regardless of expansion history. Alternatively, if neutral processes dominate, we expect genome sizes to increase during range expansion. We combine a survey of genome sizes estimated by flow cytometry with trait data collected from a common garden experiment to ask: (1) Is variation in genome size associated with developmental trait variation? (2) Are genome sizes smaller toward the leading edge of the expansion, consistent with selection for “colonizer” traits? Or alternatively, does genome size increase toward the leading edge of the expansion, consistent with expectations of founder effects and drift? (3) Finally, are genome sizes associated with elevational clines that are decoupled from the direction of expansion?

## 2 MATERIALS AND METHODS

### 2.1 Plant material

**FIGURE 1:**
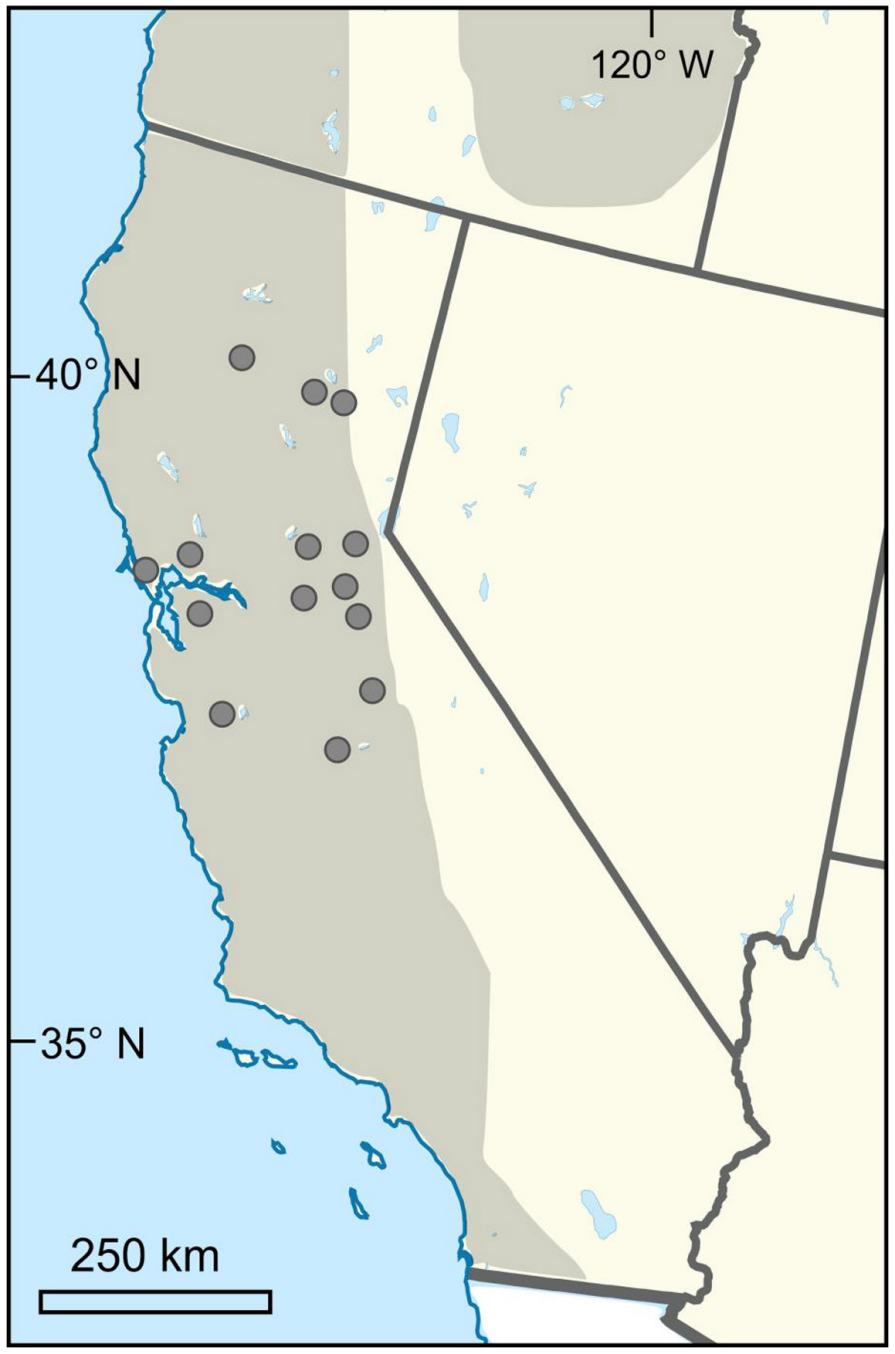
Map of 14 YST populations sampled in 2016 from the invasion in California, United States. Shaded areas indicate the invaded range in western North America.

YST is distributed patchily across California, and local populations within this invasion have been observed to vary in terms of both traits (Dlugosch et al., 2015) and effective population size (Braasch et al., 2019). Seeds were collected from 14 sites across California (Figure 1; Table 1) in August 2016 along a linear transect at each site, with maternal plants >1 m apart. Collections include populations from the coastal San Francisco Bay area, which is the putative site of introduction to California, the Central Valley where the invasion is most severe and the Sierra Nevada Mountain range, which includes the leading edge of the Californian expansion.

**TABLE 1:** Field collections from the YST invaded range in California, United States.

			Latitude, longitude	Elevation (m)	*N* (common garden)	*N* (genome size)
Coast	Diablo	DIA	N 37.863694, W 121.976444	237	20	21 (1)
	Gilroy	GIL	N 37.03373, W 121.53674	68	22	27 (2)
	Marin	MAR16	N 38.01154, W 122.612222	143	25	26 (2)
	Napa	NAP	N 38.2400167, W 122.2702545	17	22	27 (3)
Central Valley	Clovis	CLV	N 36.9231479, W 119.793704	118	17	18 (2)
	Red Bluff	RB	N 40.27083, W 122.27104	157	23	24 (1)
	Triangle	TRI	N 37.46178, W 119.79218	866	13	13
Sierra Nevadas	Arnold	ARN	N 38.262671, W 120.336713	1273	30	30 (2)
	Belden	BEL	N 40.004779, W 121.261753	679	25	26 (2)
	Lyons	LYO	N 38.078308, W 120.164527	1497	21	21 (1)
	Placerville	PLA	N 38.733866, W 120.829686	507	21	23 (2)
	Quincy	QUI	N 39.964903, W 120.944909	1075	23	24 (2)
	Sandflat	SAN	N 38.76417, W 120.327177	1192	27	29 (3)
	Vet	VET	N 38.09996, W 120.58947	386	23	24 (1)
				Total	312	333 (24)

*Note*: Sample sizes of genome size estimates for each population include the overall number of individuals with at least one measurement. Values in parentheses indicate how many individuals out of the total have replicate estimates.

### 2.2 Common garden

We collected common garden data from 13 to 30 individuals from each of the 14 populations (*N* = 312; Table 1). We germinated seeds on the surface of moist potting soil (3:2:1 ratio of Sunshine Mix #3 soil, vermiculite, and 20 grit silica sand) under fluorescent lights and 12‐h days in December 2016 and recorded germination date daily. We germinated multiple seeds from the same maternal plant and kept the first germinating individual. We transplanted all growing plants into 410 mL Deepots (Steuwe and Sons, Inc, USA) in January 2017 when they were 5 weeks old and grew them in a greenhouse at the University of Arizona in Tucson, AZ, United States. Once in the greenhouse, we randomly assigned one individual from each population to each of 30 different blocks. Additional individuals from two different maternal plants from ARN and SAN were transplanted into three and four blocks, respectively (Table 1). Plants were watered daily using an automatic drip watering system and maintained through senescence. At the rosette stage, prior to reproduction, we measured leaf number, length of longest leaf, and width of longest leaf to estimate a size index and early growth rate (as in Dlugosch et al., 2015). The size index was calculated as (leaf number*(maximum leaf length*maximum leaf width)^½^), which correlates linearly with biomass in this species (Dlugosch et al., 2015). We calculated linear growth rates using our size index and number of days since germination. The common garden was checked daily to record days to the initiation of bolting and days until the first flower. Plants were harvested when most individuals had died, after which we counted the total number of flowering heads. We dried harvested plants overnight at 60°C and weighed aboveground dry biomass.

### 2.3 Genome size estimation

We estimated genome size by flow cytometry using the FACSCanto II instrument (BD Biosciences, San Jose, CA, USA) equipped with a blue (488‐nm), air‐cooled, 20‐mW solid state laser and a red (633‐nm) 17‐mW helium neon laser for UV excitation. Sample preparation followed a modified two‐step protocol by Doležel et al. (2007) as follows. We chose *Raphanus sativus* (2C = 1.11 pg) provided by the Institute of Experimental Botany (Prague, Czech Republic) as our internal standard, as it has a close but non‐overlapping genome size with the previously reported nuclear genome size of YST (2C = 1.74 pg; Bancheva & Greilhuber, 2006). Nuclear suspensions were prepared by chopping 50–60 mg each of YST and *R. sativus* fresh tissue with 0.5 mL of ice‐cold Otto I buffer in a Petri dish on top of ice, using a sharp razor blade. The suspension was filtered first through gauze and then again through 18 μm Nylon mesh. Nuclear suspensions in Otto I buffer are considered relatively stable (Doležel et al., 2007) and samples were left covered and on ice until immediately before analysis by flow cytometry, at which point we added 0.77 mL of Otto II buffer, 29 μL of 1 mg/mL propidium iodide (PI) stain, and 7.5 μL of 1 mg/mL RNase. The sample was then gently vortexed and left for at least 5 min on ice in a covered container. YST genome sizes were calculated according to Doležel & Bartoš (2005). We recorded three estimates of genome size on different days for a subset of individuals (*N* = 24), including one to three individuals from 13 of our 14 populations. We obtained a single estimate of genome size for 13–28 additional individuals from across those 14 populations, for a total 333 individuals for which we have at least one genome size estimate (Table 1).

### 2.4 Genome size correction

Preliminary analyses of the genome size data indicated that the date of flow cytometry measurement had a significant effect on estimated genome size (see Results, Table 2). This trend might reflect variation induced by developmental changes in the plant as it ages or changes in the flow cytometer over time (Doležel & Bartoš, 2005; Doležel et al., 2007). To account for potential variation due to measurement error associated with the estimation date, we used the best‐fitting linear model predicting genome size (Table 2) to inform date‐corrected estimates that we included in subsequent trait analyses.

**TABLE 2:** Linear model explaining 2C genome size (pg) variation.

Effect type	Effect	Coefficient	df	*F*‐ratio	*p*
Fixed	**Population age**	**−0.00030**	**1**	**13.763**	**0.0002**
Fixed	**Elevation**	**−0.00001**	**1**	**3.567**	**0.0598**
Fixed	**Days until FC** ^a^	**−0.00031**	**1**	**13.022**	**0.0004**
Fixed	Population age*elevation				NS
Fixed	Population age*days until FC				NS
Fixed	Elevation*days until FC				NS
Fixed	Pop age*elevation*days until FC				NS
				*R* ^2^	0.0784
				*F*_(3,329)_	9.324
				*p*	<0.0001

*Note*: Significant effects are shown in bold. Effects without significant main or interaction effects (*p* > 0.1) were removed from the model (NS).

^a^ Number of days between germination and date of flow cytometry estimation.

### 2.5 Statistical analyses

The growth and flowering traits that we collected are likely to be correlated as part of plant development, and so we used a principal component analysis (PCA) to identify major axes of variation in these traits using base R (R Core Team, 2021). Coordinates of PC1 and PC2 were then used as response variables in linear models of the effect of genome size on traits. We fit separate linear models to predict PC1 and PC2 from fixed effects of corrected genome size, greenhouse position, days until harvesting, and interactions among all fixed effects. Greenhouse position was treated as a fixed ordinal variable, reflecting the distance from the evaporative cooling system at one end of the greenhouse, due to a temperature gradient of ~8°C across our experiment. For both analyses, model selection was performed using analyses of variance (ANOVA) to identify if the removal of non‐significant effects (*p* > 0.1) significantly improved the model.

We fit linear models to test our hypotheses that genome size declines toward the leading edge of invasion and at high elevations within the Californian invasion. Corrected genome size (*N* = 333) was modeled as a function of fixed effects of age of source population, elevation, and position in the greenhouse. We used the Jepson Online Herbarium (http://ucjeps.berkeley.edu/) for *C. solstitialis* in California to determine population age as in Braasch et al. (2019). We used the earliest occurrence record in the county of each source population, or the record for a neighboring county if the collection site was closer to an older occurrence record, as its colonization date. We found elevation using Google Earth records from latitude and longitude of collection locations. The analysis was repeated for genome size estimates that had three replicates (*N* = 24), using the mean value across replicates.

## 3 RESULTS

**FIGURE 2:**
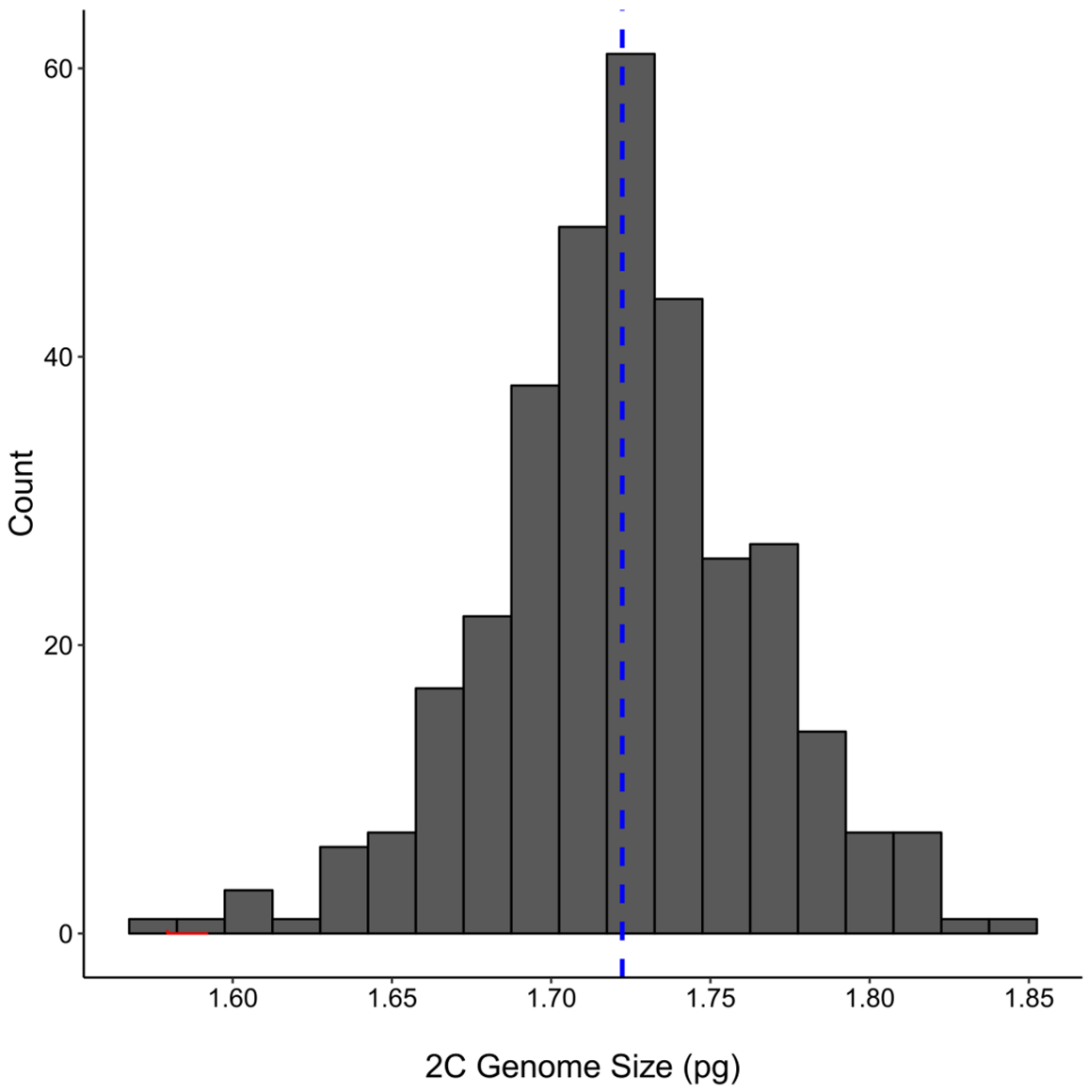
Histogram of 2C genome sizes sampled from the Californian invasion (N = 335), with a mean size of 1.72 pg (blue dashed line). For individuals with repeat measurements (N = 24), only the means of estimated genome sizes are included.

Our survey of populations in the Californian invasion identified intraspecific 2C genome size variation of 1.21‐fold across individuals, ranging from 1.58 to 1.85 pg (1.47–1.77 GB), with a mean of 1.72 pg (1.68 GB) across all genome size measurements (Figure 2). In our reduced dataset of only individuals with repeated estimates, we determined the means across each individual's estimates and recovered the same range of genome size from 1.58 to 1.85 pg (Figure S1), with a slightly greater mean of 1.74 pg (1.70 Gbp). We also found a high degree of within‐population variation in genome size, such that most populations included genome sizes spanning the range of the dataset. For example, the coastal Bay area populations MAR16 and NAP had the greatest intrapopulation range of 1.58–1.78 pg (1.55–1.74 GB) and 1.60–1.80 pg (1.56–1.76 GB), respectively, and the Central Valley population RB had the narrowest range in genome sizes at 1.66–1.76 pg (1.62–1.72 GB).

**FIGURE 3:**
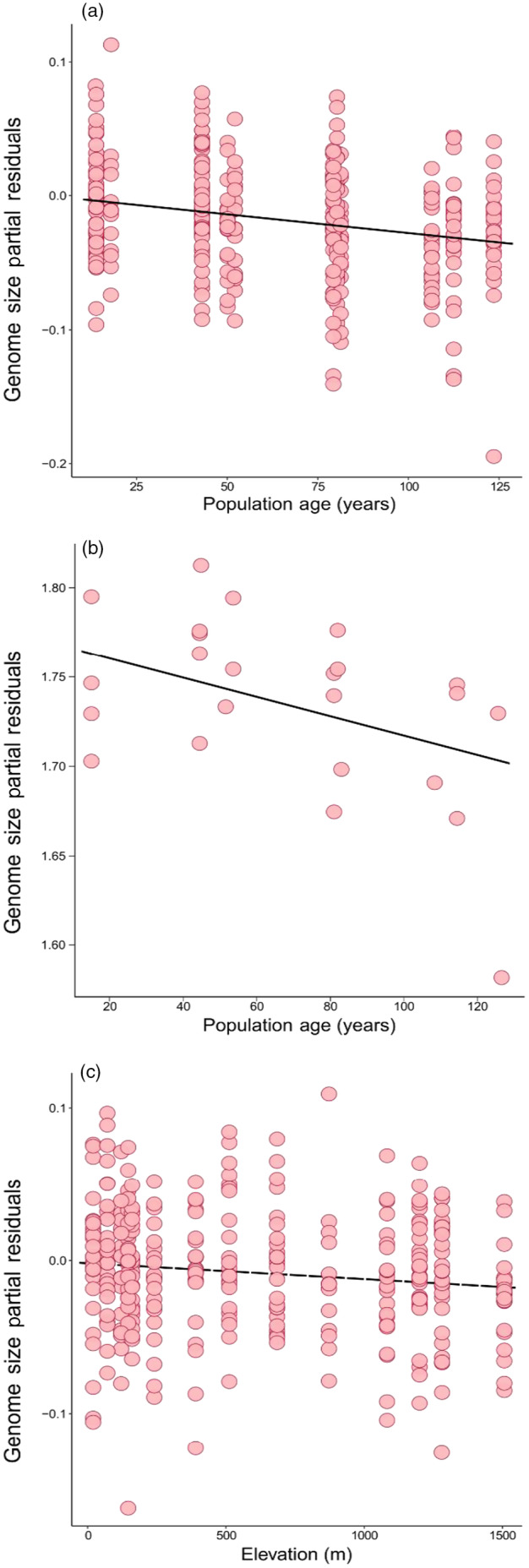
Partial residuals from best‐fitting linear models explaining patterns of genome size variation using the full genome size data set of California individuals (N = 335) by (a) population age and (c) elevation. (b) Partial residuals by population age using a reduced dataset of only individuals with repeat flow cytometry measurements (N = 24). Solid lines indicate significant associations (*p* < 0.1).

The best‐fitting linear model explaining variation in genome size identified significant negative effects of population age, such that more recently founded populations had higher genome sizes, consistent with a scenario of reduced efficacy of selection in recently established populations (Figure 3a; Table 2). The effect of elevation had a marginally significant, negative relationship with genome size, consistent with selection for faster growth and early reproduction at higher elevations, where growing seasons tend to be shorter (Figure 3c; Table 2). We repeated the analysis of genome size with 24 individuals for which we had three replicate estimates. We again found a significant negative relationship with population age (Figure 3b); however, there was no significant relationship with elevation in the reduced dataset, and no effect of genome size estimation date. Across the full dataset of genome sizes, we identified a weakly negative but significant effect of estimation date, wherein flow cytometry measurements later in the growing season were significantly smaller (𝛽= − 0.00031, *p* < 0.001). For models assessing trait variation, we applied the following correction to our raw estimates:

$$\begin{array}{c} \text{Corrected genome size}=\text{Estimated genome size}-\\ \left(-3.061\times 10^{-4}\right)\times \left(\text{Days to genome size estimation}\right). \end{array}$$

**FIGURE 4:**
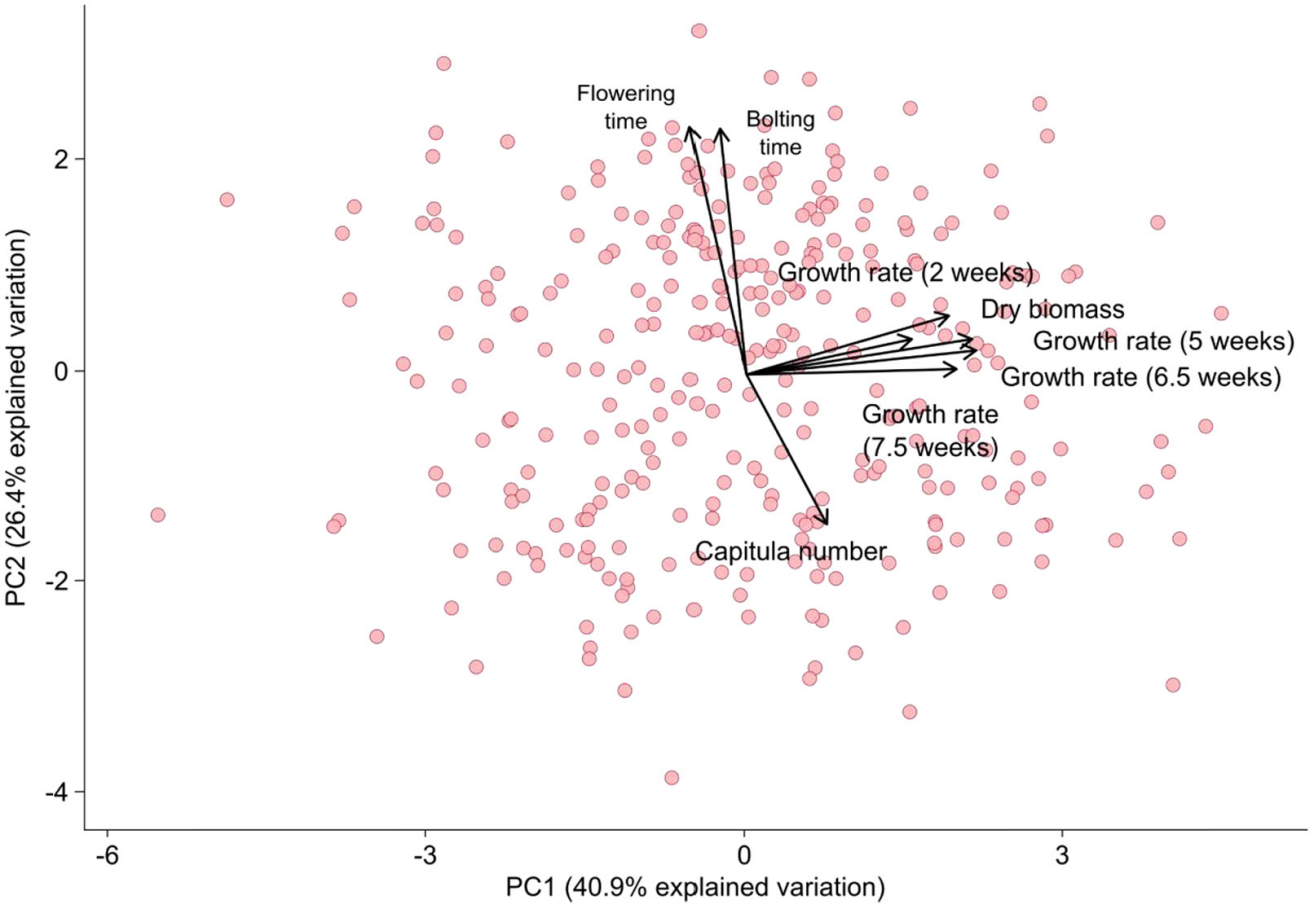
Principal component analysis of traits where each point indicates an individual plant in the common garden (N = 391) with PC1 and PC2 explaining 67.3% of all variation. PC1 is associated with variation in growth traits and PC2 is associated with variation in development an.

Among growth and flowering traits, the first two principal components explained 67.3% of total variance, with PC1 and PC2 accounting for 40.9% and 26.4% variance, respectively (Figure 4). Principal component analysis indicated that PC1 was strongly associated with growth rates and aboveground dry biomass, while PC2 was associated with bolting time, flowering time, and flower number at harvest (Figure 4). Higher PC1 values corresponded to faster growth and larger overall size. Higher PC2 values corresponded to longer time to bolting and flowering, and fewer flowers produced overall. PC1 and PC2 coordinate values were used as measures of growth and development metrics for subsequent analyses of trait variation.

**FIGURE 5:**
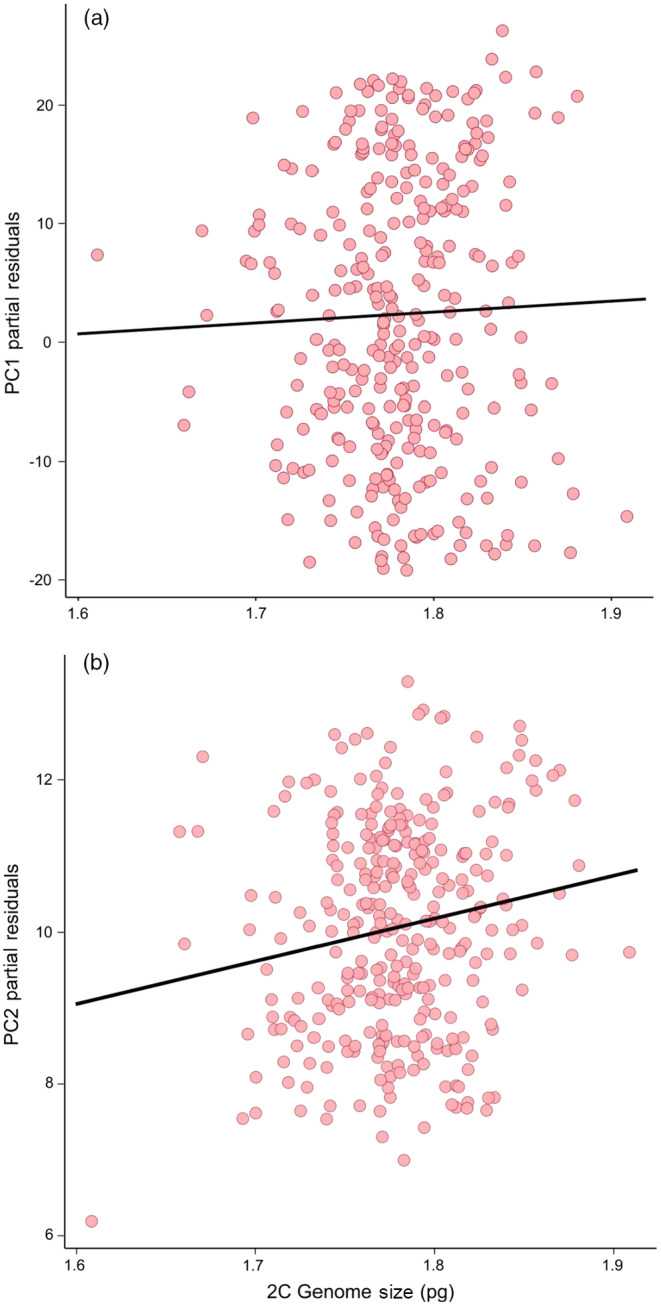
(a) Partial residual plot from best‐fitting model explaining variation in PC1 scores due to fixed effect of genome size (NS, *p* = 0.39). (b) Partial residual plot explaining variation in PC2 scores by genome size (*p* = 0.001).

**FIGURE 6:**
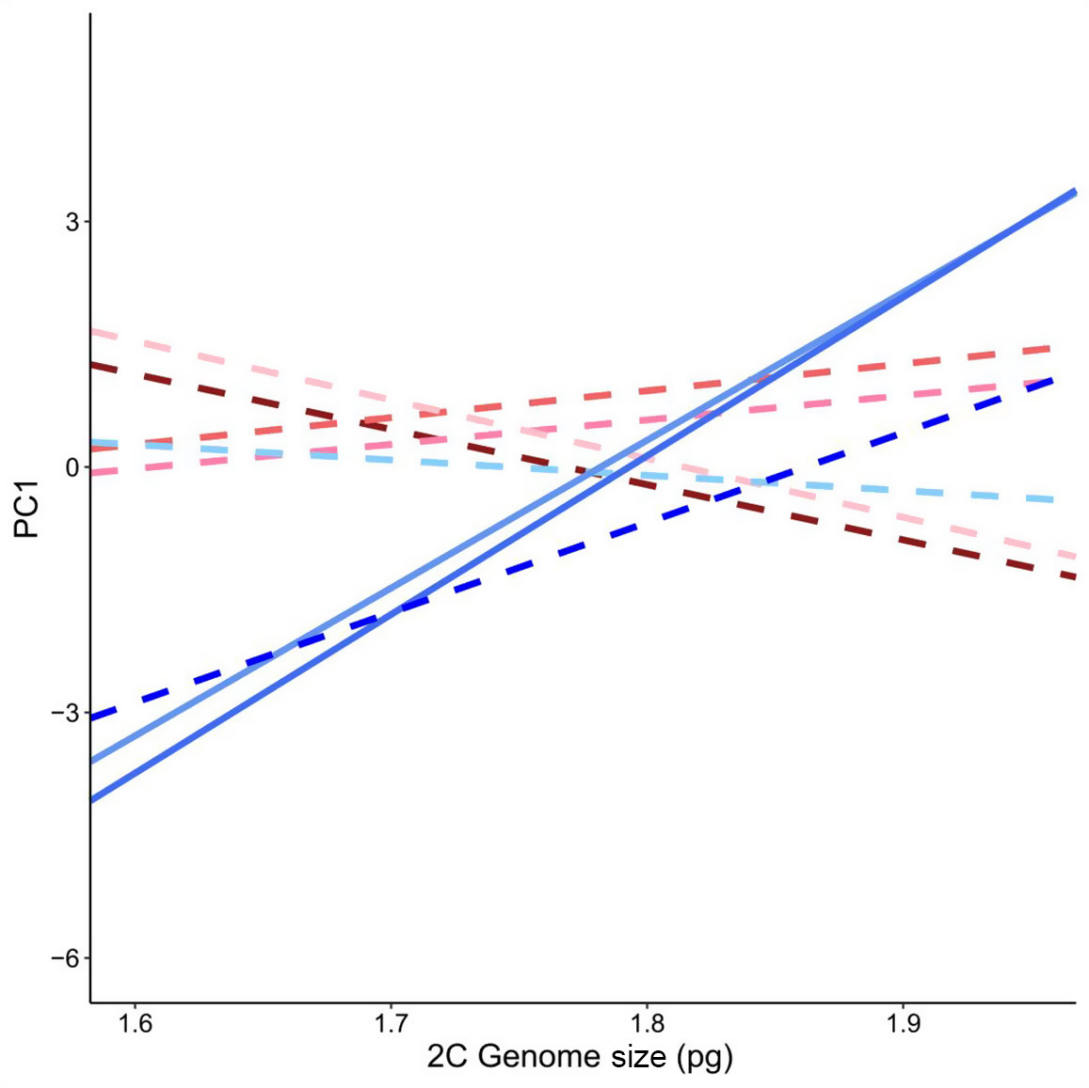
Genome size varies in direction and magnitude of effect on PC1 scores when plotted by greenhouse block. Different colour lines from red to blue indicate greenhouse position with increasing proximity to the cooling pads, where solid lines indicate a significant effect of genome size on PC1 (*p* < 0.05) Significant and marginally significant positive correlations were found in positions 7 and 8, and position 9, respectively, where local environments were coolest.

In total, we collected both flow cytometry and trait data from 312 individuals. A linear model explaining variation in PC1 scores included nonsignificant main effects of corrected genome size, with significant interactions between genome size and greenhouse position (Table 3). A partial residual plot of PC1 versus genome size shows a positive slope (Figure 5a), despite a negative coefficient predicted by the linear model on the effect of genome size alone (Table 3). This discrepancy occurred because the effect of genome size on growth‐related traits was dependent on where in the greenhouse an individual was grown (Figure 6). There were no significant effects of genome size on PC1 in positions 1–6, where environmental conditions were warmer, but positions 7 and 8 demonstrated significant associations with genome size where conditions were cooler (Table S1). We also found the direction of the relationship between genome size and growth‐related traits was more positive in blocks that were cooler, as in the case of position 9 (Figure 6). A linear model explaining variation in PC2 indicated a significant positive effect of corrected genome size (𝛽=6.476, *p* = 0.001) and negative effects of greenhouse position and its interaction with harvesting time (Table 4; Table S2). Genome size was significantly, positively correlated with the timing of reproductive events, wherein higher PC2 axis values indicate a longer time to bolting and flowering, as well as lower capitula production (Figure 5b), consistent with the prediction that genome replication costs and longer cell cycles may delay reproduction.

**TABLE 3:** Best‐fitting linear model explaining PC1 coordinates.

Effect type	Effect	Coefficient	df	*F*‐ratio	*p*
Fixed	Genome size^a^	−6.752	1	−0.857	0.392
Fixed	**Greenhouse position** ^b^	**see SI** ^c^	**8**	**3.411**	**0.0009**
Fixed	Genome size*greenhouse position	**see SI** ^c^	**8**	**2.008**	**0.0454**
Fixed	Days to harvest				NS
Fixed	Genome size*days to harvest				NS
Fixed	Greenhouse position*days to harvest				NS
Fixed	Higher order interactions				NS
Random	Block				NS
				*R* ^2^	0.126
				*F*_(17,294)_	2.481
				*p*	0.001

*Note*: PC1 coordinate values represent a composite of growth rate and biomass traits. Significant effects are in bold. Effects without significant main or interaction effects (*p* > 0.1) were removed from the model (NS).

^a^ 2C genome size corrected for effect of estimation date.

^b^ Distance to cooling pads in greenhouse, scored as positions 1 (furthest) to 9 (closest).

^c^ See Table S1 for all level and interaction coefficients.

**TABLE 4:** Best‐fitting linear model explaining PC2.

Effect type	Effect	Coefficient	*df*	*F*‐ratio	*p*
Fixed	**Genome size** ^a^	**6.476**	**1**	**3.209**	**0.001**
Fixed	**Days to harvest**	**−0.034**	**1**	**3.63**	**0.058**
Fixed	**Greenhouse position** ^b^	**see SI** ^c^	**8**	**2.09**	**0.037**
Fixed	Greenhouse position*days to harvest	**see SI** ^c^	**8**	**2.22**	**0.026**
Fixed	Genome size*days to harvest				NS
Fixed	Genome size*greenhouse position				NS
Fixed	Genome size*position*days to harvest				NS
Random	Block				NS
				*F*_(18,293)_	2.631
				*p*	<0.001

*Note*: PC2 coordinate values represent a composite of development time and flowering traits. Significant effects are shown in bold. Effects without significant main or interaction effects (*p* > 0.1) were removed from the model (NS).

^a^ 2C genome size corrected for effect of estimation date.

^b^ Distance to cooling pads in greenhouse, scored as positions 1 (furthest) to 9 (closest).

^c^ See Table S2 for all level and interaction coefficients.

## 4 DISCUSSION

Intraspecific homoploid genome size variation has been demonstrated in several plant systems, but the ecological and evolutionary factors shaping this variation, and its potential contribution of this genetic variation to species invasions, remain unclear. We conducted a survey of genome size and trait variation in YST populations across its invasion in California to test alternative eco‐evolutionary hypotheses explaining divergence in genome size during range expansion. We found that the 2C DNA content varied 1.21‐fold among all California samples, suggesting widespread and extensive genome size variation across this invasion. Genome size variation was associated with flowering time, such that plants with larger genomes reproduced later, and with lower lifetime capitula production. Genome sizes increased toward the leading edge of the invasion, but tended to decrease at higher elevations, consistent with genetic drift during range expansion but potentially strong selection for smaller genomes and faster development time at higher elevations.

Surveys of intraspecific genome size variation in other systems have documented both broad‐scale variation and conserved genome sizes across populations. For example, natural populations included 1.7‐fold variation in diploid genome size of *Brachypodium distachyon* (Oney‐Birol & Tabur, 2018), and over twofold variation in *Synura petersenii* (Čertnerová & Škaloud, 2020). Conversely, genome size can be a very stable species‐level trait with little observed variation, despite widespread population sampling (e.g., Lysák, 2000; Tuna et al., 2019). With a 1.21‐fold 2C range, YST demonstrated moderate variation on par with a previous survey in the system (Irimia et al., 2017) and with observations in several other systems including *Dactylis glomerata* (Creber et al., 1994), *Arachis duranensis* (Temsch & Greilhuber, 2001), *Lagenaria siceraria* (Achigan‐Dako et al., 2008), and *Phragmites australis* (Pyšek et al., 2018). The variation we observed in YST is also similar to systems in which significant associations between genome size and plant traits (Pyšek et al., 2018), or environmental variables (Achigan‐Dako et al., 2008; Benor et al., 2011; Bilinski et al., 2018) have been demonstrated. Our means of triplicate genome size estimates also span this range of variation, and bracket previous single estimates (Bancheva & Greilhuber, 2006; Carev et al., 2017; Miskella, 2014), indicating that measurement errors are unlikely to explain the 1.21‐fold variation observed across our samples (Greilhuber, 2005).

Notably, we detected an influence of measurement date on genome size estimates, in which later estimates tended to yield smaller genome sizes. Flow cytometry measurements that underestimate nuclear size can occur when DNA staining is inhibited (Doležel et al., 2007). Genome size measurements were taken over 5 months, during which the plants progressed from rosette formation in early development, through early senescence. Phenolic concentrations in the cytosol can vary among tissue types and across developmental stages (Wam et al., 2017; Witzell et al., 2003), some of which are known to inhibit the staining of DNA (Doležel et al., 2007). Factors that could interfere with these measurements differ across species (Doležel et al., 2007) and methodology (Loureiro et al., 2007; Noirot et al., 2003; Noirot et al., 2005; Price, 2000), which may compromise large‐scale comparisons that take many measurements over extended periods of time. Although previous work in YST found no overall differences in genome size among different parts of its global distribution (Irimia et al., 2017), an analysis accounting for changes in measurement over time does find evidence of significant differences between native and invaded ranges (Cang & Dlugosch, 2022, bioarxiv). Fortunately, our relatively large sample size allowed us to identify this change over time and most importantly, use our linear model explaining genome size variation to quantify the effect and remove it from subsequent analyses of trait variation.

Our finding that larger genomes were associated with later flowering and lower flower production joins a growing body of evidence pointing to potential developmental costs of maintaining large genomes. Across a diversity of species, genome size has been correlated with a suite of characteristics, including plant size (Carta & Peruzzi, 2016; Münzbergová, 2009), growth rate (Fridley & Craddock, 2015), pollen tube growth (Reese & Williams, 2019), seed size (Beaulieu, Moles, et al., 2007), flowering time (e.g., Benor et al., 2011; Jian et al., 2017), and rates of photosynthesis (Roddy et al., 2020). Comparative phylogenetic approaches to identifying the effects of genome size on traits have found that genome size can be a strong predictor of seed mass (Knight & Beaulieu, 2008), which may mediate the apparent effect of genome size on later developing traits (Grotkopp et al., 2004).

Strikingly, the magnitude and direction of genome size effects on growth‐related traits in our study were dependent on local environmental conditions. Our linear model demonstrated a significant effect of the interaction between greenhouse position and genome size on growth, in which genome size had a significant positive association with growth in experimental blocks that were in cooler locations. The nature of this interaction was predominantly driven by individuals with small genomes that exhibited slower growth and produced less biomass overall under cooler growing conditions. A similar effect has also been identified in *Phragmites australis* (Meyerson et al., 2020). These interactions suggest that the effect of selection on genome size, and the subsequent relationship between genome size and the traits it influences, is likely to vary across environments. For example, field surveys of an experimental grass community in the United Kingdom found that species with large genomes were underrepresented in nutrient‐poor conditions with lower productivity, but successfully competed with small genome species and produced more biomass in N + P supplemented plots (Guignard et al., 2016). Principal component analysis of traits in our study identified that major axes associated with growth and reproduction varied independently from each other, but genome size influenced variation across both axes. These relationships suggest that effects of genome size on overall plant performance and fitness are complex and can manifest across multiple independent traits and be environmentally dependent. A critical consideration for additional experiments is that the apparent scale of genome size effects on trait variation may depend on the environments under which they are measured. Variation in the strength of these effects could then be difficult to quantify without explicit testing under varying environments, and associations identified under experimental conditions might differ from the effects of genome size variation in the field.

We predicted that selection would favor smaller genome sizes in habitats that favor faster development times. There is increasing evidence of correlations between genome size and a variety of environmental conditions, such as elevation (Achigan‐Dako et al., 2008; Benor et al., 2011; Bilinski et al., 2018; Carta & Peruzzi, 2016; Díez et al., 2013), temperature seasonality (Knight & Ackerly, 2002; Paule et al., 2018; Qiu et al., 2019), atmospheric carbon (Franks et al., 2012), precipitation (Carta & Peruzzi, 2016; Knight & Ackerly, 2002), heavy metal pollution (Temsch et al., 2010; Vidic et al. 2009), and soil nitrogen (Guignard et al., 2016; Kang et al., 2015). We found marginally significant evidence for small genome sizes at high elevations in our full dataset. However, the marginally significant relationship with elevation disappeared in the reduced dataset, as did the effect of date of genome size estimation. These replicates were taken early in YST development for all individuals in the reduced dataset, and so variation in age‐specific cytosolic compound concentrations likely exerted less influence on estimation precision. The relationship between genome size and elevation is thought to be mediated by selection for rapid onset of flowering due to short growing seasons at high elevations, as appears to be the case in maize (Bilinski et al., 2018). We do observe a significant negative correlation between flowering time and elevation, such that stronger direct selection on flowering time might explain weaker patterns of genome size differentiation, given that genome size differences explain only a portion of flowering time trait variation. Our linear models cannot establish cause and effect between genome size and flowering time, however, and the fitness costs of genome size variation should be quantified in the field across elevational gradients.

In contrast, we did not find evidence that genome size declines during range expansion, as we predicted if selection favored fast development times in invaders. Rather, YST genome sizes increased toward the leading edge of the expansion, a pattern more consistent with founder effects and genetic drift contributing to the accumulation of larger genomes in populations at the leading edge. Repeated analysis of genome size with individuals for which we had three replicate estimates confirmed a significant negative relationship with population age. Previous work in this system demonstrated that more recently founded populations have lower effective population sizes, consistent with stronger genetic drift (Braasch et al., 2019). Recent theoretical work on the formation of range limits predicts that mutational load resulting from accumulation of deleterious variants during successive founding events can reduce population expansion across an environmental gradient, especially when mutations are not severely deleterious (Henry et al., 2015). A survey of genome sizes in European beech recovered similar findings, in which genome sizes increased toward range margins (Paule et al., 2018). This has particular importance for our understanding of the evolution of range limits in introduced species, as relatively strong genetic drift could allow mildly deleterious increases in genome size to accumulate and slow expansion at the leading edge. Geographic patterns of mutation accumulation in *Arabidopsis lyrata* support this prediction, in which signatures of mutational load increased toward range edges, with associated effects on individual plant performance and population growth (Willi et al., 2018). Therefore, smaller effective population size is one potential explanation for the persistence of larger genomes at the edge of expansion in spite of potential reproductive costs, and why a putatively adaptive relationship with elevation is weak. Disentangling the direction and magnitude of these potentially opposing influences on genome size will require direct estimates of selection on traits and underlying genetic polymorphisms, as well as expected population allele frequencies under neutral conditions of the sequences contributing to genome size variation.

Successful management of species invasions and prevention of future spread depends on understanding the factors that shape the dynamics of range expansion. There is a long history in invasion biology of seeking traits that are associated with invaders, but we have only recently begun to investigate structural genomic variation (Baduel et al., 2019; Bilinski et al., 2018; Goubert et al., 2017; Kuester et al., 2014; Pyšek et al., 2018; Schrader et al., 2014; Stapley et al., 2015). Our data contribute to increasing evidence that intraspecific genome size variants are common and could generate genetic and phenotypic variation on very short timescales, which may be important to establishment and range edge formation during invasions.

## CONFLICT OF INTEREST STATEMENT

The authors declare no conflict of interest.

## Supporting information


Data S1.

## Data Availability

Data for this study are available at: to be completed after manuscript is accepted for publication.
