# Supplementary material for: Genome size variation and evolution during invasive range expansion in an introduced plant
Source: Evol Appl. 2023 Dec 11;17(1):e13624. doi: 10.1111/eva.13624 (PMC10810172; doi:10.1111/eva.13624)
Supplement: Supplementary file 1 — Data S1. [file EVA-17-e13624-s001.pdf]

Supplementary Tables and Figures

Table S1. Coefficient details for full best-fitting linear model explaining PC1

Effect type	Effect	Coefficient	t-value	*p*
Intercept		11.938	0.847	0.398
Fixed	genome size[a]	-6.752	-0.857	0.392
Fixed	greenhouse position 2[b]	0.699	0.039	0.969
Fixed	greenhouse position 3	-16.914	-0.940	0.348
Fixed	greenhouse position 4[b]	-16.731	-0.761	0.447
Fixed	**greenhouse position 5[b]**	1.079	0.056	0.956
Fixed	greenhouse position 6	-8.670	-0.486	0.627
Fixed	greenhouse position 7[b]	**-44.128**	**-2.374**	**0.018**
Fixed	**greenhouse position 8[b]**	**-46.704**	**-2.179**	**0.030**
Fixed	greenhouse position 9[b]	-32.402	-1.709	0.089
Fixed	genome size*greenhouse 2	-0.056	-0.006	0.996
Fixed	genome size*greenhouse 3	10.034	0.994	0.321
Fixed	genome size*greenhouse 4	9.734	0.791	0.430
Fixed	genome size*greenhouse 5	-0.422	-0.039	0.969
Fixed	genome size*greenhouse 6	4.880	0.488	0.626
Fixed	**genome size*greenhouse 7**	**24.818**	**2.384**	**0.018**
Fixed	**genome size*greenhouse 8**	**26.143**	**2.181**	**0.030**
Fixed	genome size*greenhouse 9	17.743	1.674	0.095
Fixed	days to harvest			NS
Fixed	genome size*days to harvest			NS
Fixed	greenhouse*days to harvest			NS
Fixed	higher order interactions			NS
Random	block			NS
			R^2	0.126
			$F_{(17,,294)}$	2.481
			p	0.001

PC1 coordinates represent a composite of growth-related traits. Significant effects are bold, and effects without significant main or interaction effects were removed.
[a] 2C genome size corrected for effect of estimation date
[b] distance to cooling pads in greenhouse, scored as positions 1(furthest) - 9 (closest)

Table S2. Coefficient details for full best-fitting linear model explaining PC2

Effect type	Effect	Coefficient	t-value	*p*
Intercept		-1.253	-0.185	0.853
Fixed	**genome size[a]**	**6.476**	**3.209**	**0.001**
Fixed	greenhouse position 2[b]	-12.131	-1.676	0.095
Fixed	greenhouse position 3	2.333	0.269	0.788
Fixed	greenhouse position 4[b]	-14.497	-1.946	0.053
Fixed	**greenhouse position 5[b]**	**-21.397**	**-2.736**	**0.007**
Fixed	greenhouse position 6[b]	-12.138	-1.170	0.243
Fixed	greenhouse position 7[b]	-19.657	-1.914	0.057
Fixed	**greenhouse position 8[b]**	**-30.469**	**-2.779**	**0.006**
Fixed	greenhouse position 9[b]	-9.713	-0.978	0.329
Fixed	days to harvest	-0.034	-1.904	0.058
Fixed	greenhouse 2*days to harvest	0.039	1.669	0.096
Fixed	greenhouse 3*days to harvest	-0.007	-0.236	0.813
Fixed	**greenhouse 4*days to harvest**	**0.048**	**2.017**	**0.045**
Fixed	**greenhouse 5*days to harvest**	**0.071**	**2.787**	**0.006**
Fixed	greenhouse 6*days to harvest	0.040	1.189	0.235
Fixed	**greenhouse 7*days to harvest**	**0.066**	**1.986**	**0.048**
Fixed	**greenhouse 8*days to harvest**	**0.102**	**2.856**	**0.005**
Fixed	greenhouse 9*days to harvest	0.034	1.062	0.289
Fixed	genome size*days to harvest			NS
Fixed	genome size*greenhouse			NS
Fixed	higher order interactions			NS
Random	block			NS
			R^2	0.139
			$F_{(18,293)}$	2.631
			p	<0.001

PC2 coordinate values represent a composite of development-related traits. Significant effects are shown in bold, and effects without significant main or interaction effects were removed.

[a] 2C genome size corrected for effect of estimation date

[b] distance to cooling pads in greenhouse, scored as positions 1(furthest) - 9 (closest)

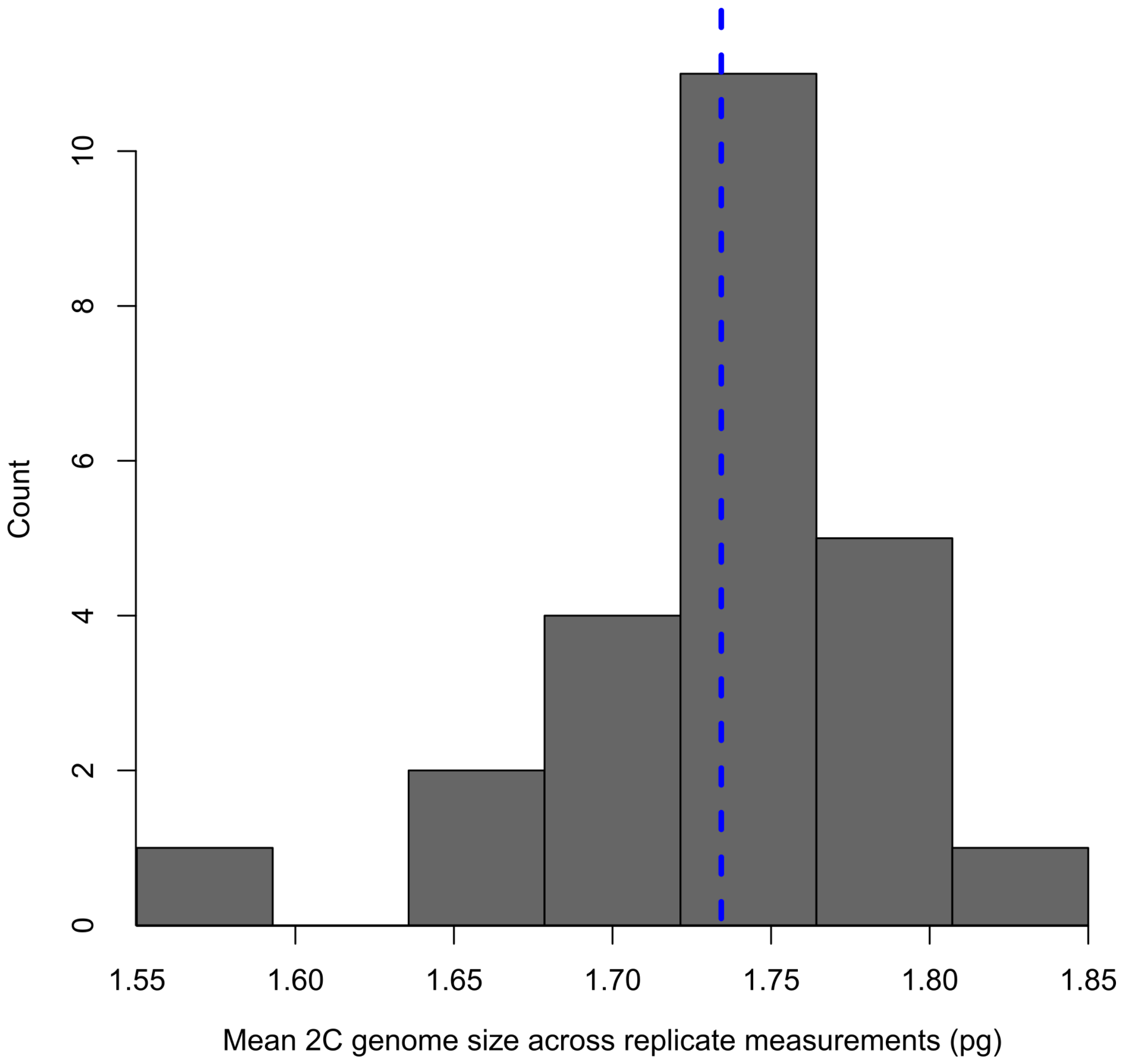

Figure S1. Distribution of within-individual mean genome sizes, including only those that had 3 replicate measurements (N=24). Dashed line is the mean genome size across individuals (mean=1.734pg).
